# Ellipse method for measuring Liaw’s anteversion of the acetabular component after total hip arthroplasty

**DOI:** 10.1186/s12891-020-03669-5

**Published:** 2020-10-08

**Authors:** Kuei-Lin Yeh, Tai-Yin Wu, Hsuan-Hsiao Ma, Sheng-Mou Hou, Chen-Kun Liaw

**Affiliations:** 1 Department of Orthopedics, Shin Kong Wu Ho-Su Memorial Hospital, Taipei, 11101 Taiwan; 2 Department of Family Medicine, Zhongxing Branch, Taipei City Hospital, Taipei City, 10341 Taiwan; 3 Institute of Epidemiology and Preventive Medicine, National Taiwan University, Taipei City, 10055 Taiwan; 4 National Taipei University of Nursing and Health Science, Taipei City, 11219 Taiwan; 5 Department of Orthopedics and Traumatology, Taipei Veterans General Hospital, Taipei, 11217 Taiwan; 6 Department of Orthopedics, School of Medicine, National Yang-Ming University, Taipei, 11221 Taiwan; 7 Department of Orthopedics, Shin Kong Wu Ho-Su Memorial Hospital, Taipei, 11101 Taiwan; 8 Department of Orthopedics, School of Medicine, College of Medicine, Taipei Medical University, Taipei City, 11031 Taiwan; 9 Department of Orthopedics, Shuang Ho Hospital, Taipei Medical University, No. 291, Zhongzheng Rd., Zhonghe Dist, New Taipei City, 23561 Taiwan; 10 Graduate Institute of Biomedical Optomechatronics, College of Biomedical Engineering; Research Center of Biomedical Device, Taipei Medical University, Taipei City, 11301 Taiwan

**Keywords:** Anteversion measurement, Ellipse method, Total hip arthroplasty, Liaw’s anteversion

## Abstract

**Background:**

Several cup anteversion measurements for post-operative total hip arthroplasty (THA) surgery have been described. We developed the standardized Liaw’s trigonometric method to eliminate the influence of patient position, which is the most accurate method for cup anteversion measurement. We then developed an ellipse method using the Elliversion software and reported an interobserver error reduction in 2013. In this study, we attempted to apply this ellipse method in the clinic and compared its accuracy with the standard trigonometric version.

**Methods:**

In the present study, we attempted to incorporate the ellipse method with Liaw’s standardized anteversion in the simulated cup position. We measured standardized Liaw’s anteversion for 434 radiographs in the clinic using the ellipse method. Repeated standard deviation (RSD) was calculated for accuracy evaluation. Furthermore, paired t-test was used for comparison with the interobserver and intraobserver errors.

**Results:**

We found that the standardized Liaw’s anteversion measured using the ellipse method showed lower RSD than the radiographic version. RSD was 0.795 in the standardized Liaw’s anteversion with ellipse method group. The *p*-values of the paired t-test were all higher than 0.05 when measuring the interobserver and intraobserver errors. It indicated that the ellipse method was a precise and simple tool for cup anteversion measurement.

**Conclusion:**

We believe that this ellipse measurement can assist surgeons while placing the acetabulum cup into the precise position and enable early diagnosis of acetabulum loosening.

## Background

With today’s aging society, primary and revision total hip arthroplasty (THA) surgeries are expected to increase over the next 20 years [1]. Implant stability and cup loosening will affect a large number of patients with clinical presentations, such as hip pain and limited range of motion (ROM). Obvious cup loosening with enormous anteversion changes could be judged by radiographs. However, in cases of early cup loosening, the changes in the acetabular version are always small. Thus, the development of a precise measure to detect small changes in acetabular version would aid in the early detection of cup loosening [2].

Proper cup orientation plays an important role in THA longevity, ROM, and rate of dislocation [2, 3]. Abduction and anteversion are two important factors that determine orientation. The abduction angle is measured at the intersection of a line drawn along the long axis of the cup with a line drawn through the horizontal line of the pelvis. Cup anteversion can be measured in several different ways, including Fabeck’s method, Pradhan’s method, Widmer’s method and Liaw’s trigonometric method [4]. In addition, commercially available software such as OrthoView and TraumaCad are capable of measuring acetabular version; however, their application is often impractical due to high cost.

Computed tomography (CT) can also be used to measure cup anteversion [5]. However, due to the cost, radiation exposure, and low resolution obtained with CT [6], this method is seldom used to monitor cup positioning after THA in most hospitals, including ours. Plain pelvic radiography is generally considered a more practical, low-cost, and low radiation-exposure method. Unlike CT-assisted methods, the accuracy of radiography-based methods largely depends on patient positioning during radiograph acquisition. Therefore, we developed Liaws trigonometric method to minimize the effect of patient positioning by accounting for variations in pelvic tilt, pelvic rotation, and component inclination. Several published studies have also validated the Liaws trigonometric method and found it to have a higher accuracy than other methods, such as Fabeck’s method, Pradhan’s method, Lewinnek method, Hassan method, and Widmer’s method [7, 8].

Trigonometric methods were the first methods developed for evaluating the Liaw’s anteversion. When viewing post-THA radiographs, the cup implant is obscured by the femoral head in most cases. In the absence of assisting lines, it is difficult to identify the endpoints of long axes and short axes, and if the examiners do not specialize in hip anatomy and radiographs, interobserver error may occur. Therefore, we developed the ellipse method in 2013 to measure Liaw’s anteversion [9]. In this study, we used the ellipse method to measure Liaw’s anteversion in a clinical setting and compared its accuracy with the standard trigonometric version. Our goal is to create a simple, reliable, and accurate method to measure anteversion precisely to aid in surgical decision-making regarding appropriate timing of THA revision.

We evaluated the accuracy of radiographic and Liaw’s anteversion measurements using the ellipse method. The accuracy was determined using repeated standard deviation (RSD). The lower the value of RSD, the more accurate the method. By calculating the RSD, the most accurate anteversion measurement method can be determined.

## Methods

### Subjects

All pelvic radiographs from patients who underwent THA in Shin Kong Wu Ho-Su Memorial Hospital were retrospectively reviewed from September 2016 to September 2018. Exclusion criteria included poor quality radiographs (e.g., unclear pubic symphysis, teardrop, or sacrococcygeal junction), lack of at least two pelvic radiographs, obvious prosthetic cup loosening, history of cup loosening history, and previous revision THA surgery. This study was approved by the institutional review board of Shin Kong Wu Ho-Su Memorial Hospital, Taiwan (number 20181005R, Approval date: 2018/11/08).

### Anatomic landmarks on radiographic images

With the ellipse method, radiographic landmarks play an important role and include the pubic symphysis, tear drop, and sacrococcygeal junction. The vector from the center of the sacrococcygeal junction to the upper pole of the symphysis pubis in the midline determines the radiographic pelvic axis. The line drawn through the bilateral teardrops is defined as the trans-teardrop line.

### Anteversion equation

Both radiographic anteversion and Liaw’s trigonometric anteversion were calculated for each patient in this study. Radiographic anteversion was defined by the equation of McLaren [10]:

$$ \mathrm{Anteversion}=\mathrm{arc}\ \sin\ \left(\mathrm{short}\ \mathrm{axis}/\mathrm{long}\ \mathrm{axis}\right) $$

Liaw’s anteversion was defined as follows:

$$ \mathrm{Anteversion}=\mathrm{arc}\ \sin\ \left(\tan\ \upbeta \mathrm{p}\right) $$

**Fig. 1:**
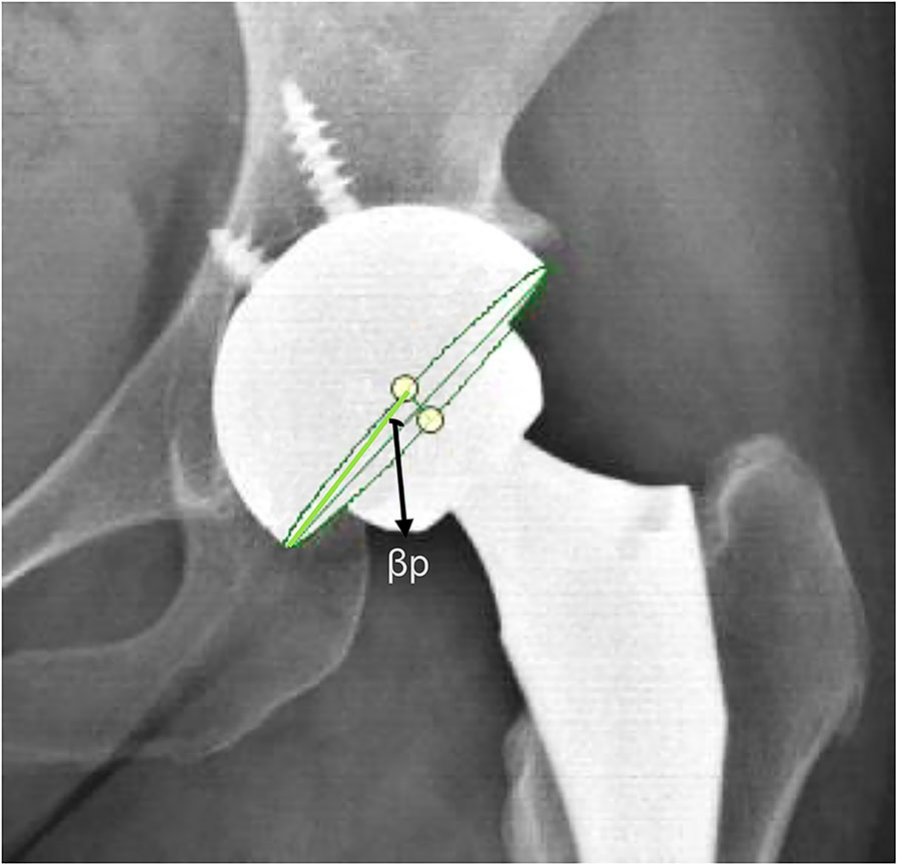
Liaw’s anteversion = arc sin (tan βp). Angle βp is the angle between the major axis of ellipse, and the two endpoint connection of the major and minor axes

The angle βp is the angle between a vector connecting the endpoints of the major and minor axes and the major axis of the component (Fig. 1).

The standardized Liaw’s trigonometric method was defined as follows:

$$ \mathrm{Anteversion}= arc\;\mathit{\sin}\;{\left(\frac{-h\times \mathit{\sin}\varphi \times \mathit{\cos}\theta +v\times \mathit{\cos}\varphi \times \mathit{\cos}\theta +\mathit{\sin}\theta \times \left({ssd}^2-{h}^2-{v}^2\right)}{ssd}\right)}^{0.5} $$

In this equation, *ssd* is defined as the length from the upper pole of the symphysis pubis to the sacrococcygeal junction; *h* represents the horizontal displacement of the sacrococcygeal junction related to the upper pole of the symphysis pubis. The *h* is positive if the sacrococcygeal junction is between the acetabulum and the upper pole of symphysis pubis; otherwise, it is negative. The vertical displacement of the sacrococcygeal junction related to the upper pole of the symphysis pubis is defined as *v*. Similar to *h*, if the sacrococcygeal junction is above the upper pole of the symphysis pubis, *v* is positive. The *u* represents the radiographic (planar) anteversion angle. The inclination (abduction) angle is defined as *w* [7].

### Software

Elliversion software was used to measure anteversion from Digital Imaging and Communications in Medicine files imported from our picture archiving and communication system (PACS). The software allows the user to apply an ellipse to the acetabulum and adjust it by altering the long and short axes. Since the curves cannot be drawn directly, the ellipse is separated into 64 parts (i.e., the ellipse is formed by 64 lines, and drawn as a 64-sided polygon). The endpoint of each line is calculated by the position and length of the long and short axes.

**Fig. 2:**
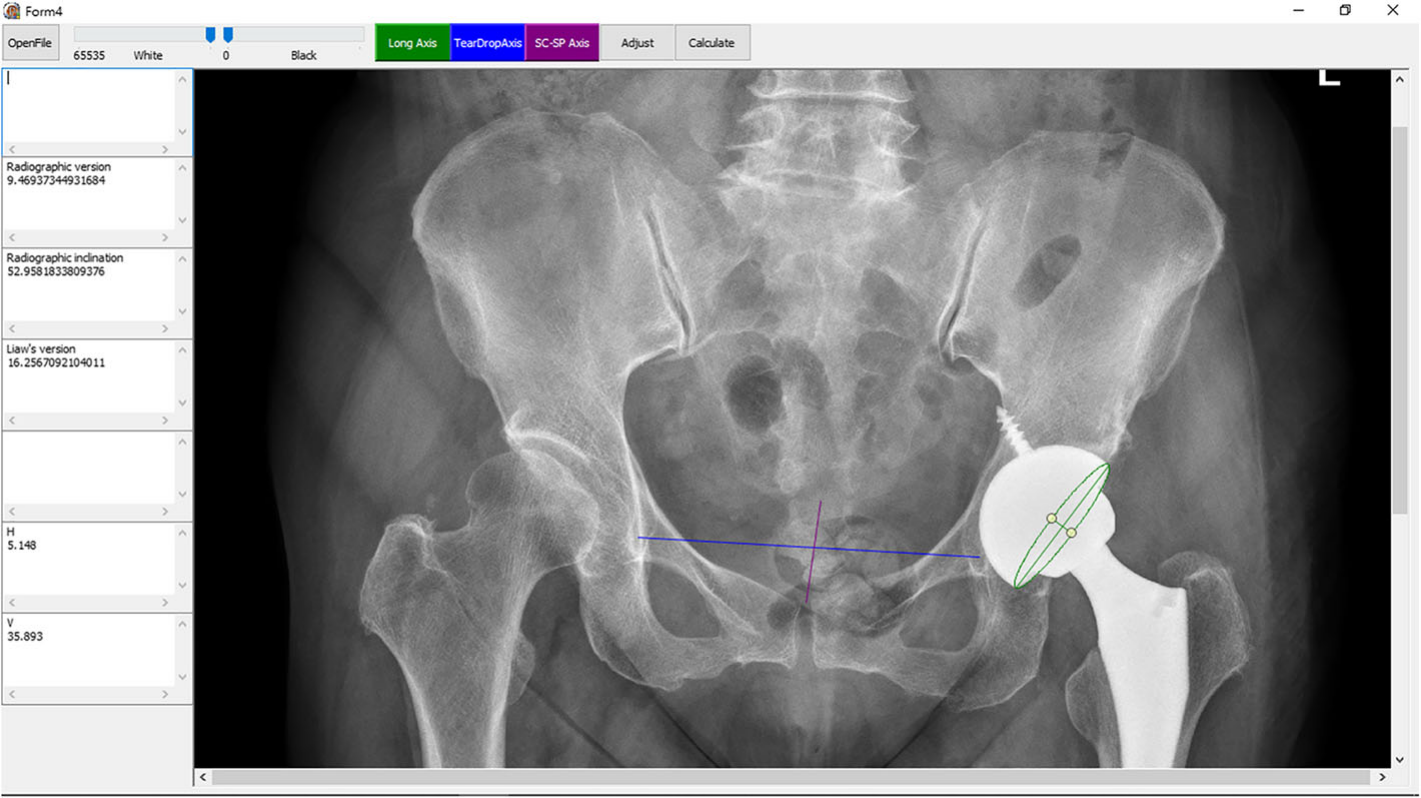
Ellipse method to measure the cup anteversion. The green line represents the cup margin of the cup, which is drawn by adapting the major and minor axes. The purple line represents the radiographic pelvic axis. The blue line represents the trans-teardrop line

The entire ellipse was drawn by adapting the major axis and minor axis to match the ellipse with the cup implant image. In addition, the trans-teardrop line and the pelvic axis were drawn, followed by calculation of both radiographic and Liaw’s anteversion (Fig. 2).

### Statistical analysis

Repeated standard deviation was used to compare radiographic and Liaw’s anteversion. Lower RSD corresponds to greater precision. RSD was calculated using the following equation:

$$ RSD=\sqrt{\frac{\sum_{j=1}^c{\sum}_{i=1}^n{\left({X}_{ij}-{\overline{X}}_j\right)}^2}{\sum_{j=1}^c\left({n}_j-1\right)}} $$

X_ij_ represents the result of the j^th^ patient and the i^th^ radiographic measurement. Xj represents the mean result of the j^th^ patient and n_j_ represents the total number of measurements of the j^th^ patient.

All anteversion measurements for each patient were performed by two different observers. Paired t-test was used to analyze inter- and intraobserver measurement differences. Statistical analysis was performed using SPSS software, version 1.0.0.1174 (IBM Corp., Armonk, NY, USA).

## Results

We analyzed 434 radiographic images from 105 postoperative THA hips in 82 patients (53 women, 29 men; age range: 28–86 years). Of the 82 patients, 23 underwent bilateral THA. Tonnis grade II or III hip osteoarthritis was the indication for surgery in 54 patients; 28 underwent surgery for Ficat stage III or IV avascular necrosis of the femoral head.

The RSDs of the radiographic and Liaw’s anteversion measurements were 1.802° and 0.795°, respectively (Table 1). Thus, Liaw’s anteversion seems to be more precise. There were no significant interobserver differences in the standardized Liaw’s anteversion measurement group (*p* = 0.325) or radiographic anteversion group (*p* = 0.644). Intraobserver differences in each group were not significant either (*p* = 0.488 and *p* = 0.502, respectively).

**Table 1:** Repeated standard deviation of the anteversion methods of measurement

	RSD
Standardized Liaw’s anteversion using the ellipse method	0.795
^a^Standardized Liaw’s anteversion without the ellipse method	0.99
Radiographic anteversion using the ellipse method	1.802

^a^data from our previous 2008 study [7]

*RSD* repeated standard deviation

## Discussion

Long-term outcome of THA is closely related to the orientation of the implanted cup. Cup movement and malpositioning can cause impingement, dislocation, limited ROM, and increased polyethylene wearing post-operatively [11–14]. Plain radiographs are widely used for clinical follow-up and assessment of cup angular position. They are practical for clinical use due to low cost and minimal radiation exposure. Anteversion and inclination are relevant measurements that evaluate cup position. Although cup inclination is easily measured, anteversion can be difficult to ascertain from pelvic radiographs.

For some orthopedic surgeons, the clinical value of measuring acetabular anteversion precisely is doubtful. However, precise measurements can detect small but significant cup movement and possibly minimize the incidence of cup loosening and prosthetic hip dislocation. Until recently, there has been no precise cup anteversion measurement. In addition, the optimal anteversion angle remains controversial. Although a previous study claimed that there is no absolute ideal anteversion angle, most surgeons recommend between 5° and 30° [15]. Another important factor related to THA revision is change in acetabular component orientation [16, 17]. For accurate evaluation of acetabular anteversion, CT examination or cross-table lateral radiography is required.

Several previous studies have reported that implanted cup measurements can be calculated using CT or plain radiography [18, 19]. The CT-assisted method is recommended to measure cup orientation owing to its good inter- and intraobserver reliability [6]. In addition, it has a high level of precision regardless of patient positioning during image acquisition [20]. However, practicality in clinical use is an issue due to its high cost and level of radiation exposure. Radiation exposure is 16.7 times higher for pelvic CT (10 mSV) than pelvic radiography (0.6 mSV). In addition, according to 2019 Taiwan Health Insurance, the cost is 13.3 times higher (pelvic X-ray, NT450, about $14.7 USD; pelvic CT, NT6000, about $196.5 USD) (http://www.nhi.gov.tw)). Thus, the benefit of plain radiography over CT is obvious [20]. After THA, we routinely follow plain pelvic radiographs longitudinally and compare the films over time. Radiographs are readily available, consistent, reproducible, economical, quick, and easy to interpret [21].

Cup anteversion is difficult to measure for several reasons. Patient positioning during film acquisition affects the measurement due to variations in pelvic tilt, pelvic rotation, and component inclination. Accuracy depends on using standardized patient positions. Additionally, due to recent advances in prosthetic materials, there are many different types of liners, such as ceramic, metallic, and polyethylene. Presence of a ceramic or metal liner may cause difficulty in measuring the edge of the acetabular component. Nonetheless, there are numerous methods of measurement [1, 4, 22]. Some studies have determined anteversion angle with fluoroscopy or multiple radiographs. The reliability and accuracy of these various radiological methods have not been studied in depth and previous studies have shown conflicting results [3, 23, 24]. However, Liaw’s method currently has the smallest error rate [1, 25]. In this study, RSD was lower for Liaw’s method than the radiographic method, indicating better accuracy.

In our previous study, the RSD of Liaw’s anteversion without using the ellipse method was 0.99° [7]. In this study, we used the ellipse method and found that the RSD was 0.795°, an even better precision, indicating better accuracy obtained using the ellipse method. In addition, two times of RSD represented the meaningful and significantly different data within the 95% confidential interval. Thus, once the anteversion we measured is 1.59° different from average anteversion on serial pelvic radiographs, it indicates significant change in cup anteversion. Detecting small changes in anteversion on plain radiographs can help alert regarding cup loosening.

Clinically, obvious cup loosening might present as hip pain and may end up being treated with revision cup surgery. However, early cup loosening might be related to excessive hip ROM, implant impingement, or insufficient bony ingrowth in the implants. When patients complain about hip pain, cup loosening might be detected only by the ellipse method with Liaw’s anteversion. Early and tiny cup loosening might be indicated. Thus, we can ask the patient to eliminate the amplitude of hip ROM and the duration of weight bearing even while discussing revision THA surgery. Although CT examination is a common and standard preoperative evaluation for the detection of cup loosening, it is difficult to identify the cause of all postoperative hip pain without any radiographic evidence of cup loosening. Without the assistance of the ellipse method, we might ignore a slight loosening of the cup.

In recent studies, an increasing number of computer-assisted orthopedic systems to guide placement of prosthetic components in THA have been described [26]. Imageless navigation was developed in 2006 and has proved to be as reliable as CT-assisted navigation for positioning the acetabular component. However, previous navigation system studies have only focused on position of the implanted cup during implantation. Postoperative position has not been studied due to lack of an accurate method to detect small amounts of cup movement. However, precise position measurement can be performed now and it is time to re-evaluate its importance. In addition, our results showed no significant interobserver and intraobserver differences for standardized Liaw’s anteversion measurements. Our findings indicate reliability, simplicity, and precision of the ellipse method.

Some researchers may question why we merely compared Liaw’s method with the radiographic version instead of other anteversion measurements. The reason is that only the standardized Liaw’s anteversion measurement corrects for patient positioning during film acquisition. Without standardizing, the accuracy of measurement is similar. Thus, we chose radiographic anteversion as defined by McLaren as the comparison [10].

The accuracy of our method for measuring anteversion has been discussed in several previous studies, which revealed a mean anteversion difference of 4.1° between Liaw’s method and CT measurement (*p* < 0.001) [3]. In some cases, a ceramic or metal liner was used, which caused the acetabular component border to appear ambiguous and cup identification difficult on radiographs. Thus, defining the apex of the ellipse was difficult. If the border of the implanted cup on radiography is not clear, anteversion cannot be accurately measured by Liaw’s method. However, the ellipse method corrects this shortcoming.

Though the ellipse method seems to be impeccable, there are some limitations. A high quality pelvic anteroposterior radiograph is required. In addition, the images should have clear delineation of the pubic symphysis, sacrococcygeal junction, as well as the teardrops bilaterally. If these anatomic landmarks are not clearly visible, the ellipse method cannot be used.

Trauma CAD can detect anteversion regardless of the position of pelvis and is available for clinical use in some hospitals. We believe that the precision of this technique will be similar to that of the ellipse method, and the precision can be further improved using Liaw’s anteversion formula. We may undertake a future study to verify this point after trauma CAD becomes available.

Our aim is to promote the use of a simple and accurate ellipse method in clinical practice. We will provide the software online for clinical use. We hope that the ellipse method will help in the early detection of cup-loosening.

## Conclusion

In conclusion, the standardized Liaw’s anteversion measurement using the ellipse method is the most precise anteversion measurement to date. The method is simple, reliable, and precise. Change in anteversion > 1.59° on serial pelvic radiography indicates cup movement and allows detection of early cup loosening. The early detection of tiny cup loosening may help to understand the unknown cause of postoperative hip pain. The early detection of cup loosening would warn the patient against excessive range of motion, or may be a signal for the need of a cup revision.

## Data Availability

The datasets used and analyzed during the current study are available from the corresponding author on reasonable request.

## Abbreviations

THA: Total hip arthroplasty
RSD: Repeated standard deviation
ROM: Range of motion
CT: Computed tomography
PACS: Picture archiving and communication system
